# Inequality in iron and folic acid consumption and dietary diversity in pregnant women following exposure to maternal nutrition interventions in three low- and middle-income countries

**DOI:** 10.1017/S1368980024001150

**Published:** 2024-05-24

**Authors:** Deepali Godha, Sandra Remancus, Tina Sanghvi

**Affiliations:** 1 Consultant for Alive & Thrive Initiative, FHI Solutions, FHI 360, Indore, MP, India; 2 Alive & Thrive Initiative, FHI 360, Washington DC, USA

**Keywords:** Wealth inequality, Education inequality, Erreygers index, Maternal nutrition intervention programmes, Bangladesh, Burkina Faso, Ethiopia, Adequate IFA consumption, Women’s dietary diversity, Counselling on IFA and Dietary diversity

## Abstract

**Objective:**

Research is available on improved coverage and practices from several large-scale maternal nutrition programmes, but not much is known on change in inequalities. This study analyses wealth and education inequality using Erreygers and Concentration indices for four indicators: adequate iron and folic acid (IFA) consumption, women’s dietary diversity, and counselling on IFA and dietary diversity.

**Design:**

A pre-test–post-test, control group design.

**Setting:**

Maternal nutrition intervention programmes conducted in Bangladesh, Burkina Faso and Ethiopia during 2015–2022.

**Participants:**

Recently delivered women (RDW) and pregnant women (PW).

**Results:**

Statistically significant reductions in education inequality were observed for adequate IFA consumption, counselling on IFA and dietary diversity in intervention areas of Bangladesh and for adequate IFA consumption in intervention areas of Burkina Faso.

A significant decrease in wealth inequality was observed for adequate IFA consumption in the intervention areas of Bangladesh, whereas a significant increase was observed in the non-intervention areas for counselling on IFA in Ethiopia and for dietary diversity in Burkina Faso.

**Conclusion:**

The results can be attributed to the extensive delivery system at community level in Bangladesh and being predominantly facility-based in Burkina Faso and Ethiopia. COVID-19 disruptions (in Burkina Faso and Ethiopia) and indicator choice also had a role in the results.

The main takeaways for nutrition programmes are as follows: (a) assessing inequality issues through formative studies during designing, (b) monitoring inequality indicators during implementation, (c) diligently addressing inequality through targeted interventions, setting aside resources and motivating frontline workers to reduce disparities and (d) making inequality analysis a routine part of impact evaluations.

Several large-scale maternal^(1)^ and child^(2)^ nutrition programmes have been successfully integrated into public health systems and child development initiatives. While these programmes were evaluated on nutrition coverage, impact, cost-effectiveness and implementation issues like integration and scaling-up^(2–4)^, the extent to which they affected wealth and education inequality is not known. The literature on poverty and health equality shows that despite aggregate improvements in maternal and child health and nutrition coverage and practices, disparities among groups often continue and may even widen^(4)^. A review of nutrition interventions, including those for pregnant women (PW), integrated into public health systems in low- and middle-income countries, particularly nutrition education and counselling (twenty-five studies) and micronutrient supplementation (forty-seven studies) lamented the ‘lack of attention to equity of intervention coverage’^(5)^. The Global Nutrition Report 2020 asserts that achieving Sustainable Development Goals (SDGs) and the 2025 global nutrition goals will require both multifaceted and equitable nutrition interventions^(6)^. The aforementioned study and report speak of wealth equity, a term that indicates equal utilisation across wealth groups standardised by need. Wealth equality, on the other hand, indicates equal utilisation across wealth groups without the standardisation by need. This study utilised equality (rather than equity) measures to understand and improve the design and implementation of nutrition programmes for reducing inequality.

The few studies that have looked at disparities in nutrition programmes have focused on the broader issue of nutritional status rather than intervention coverage^(7–11)^ Most of the previous studies have used the slope index of inequality and relative index of inequality to analyse inequality. Of the two regression-based indices, slope index of inequality measures the absolute change in health level between the most and the least disadvantaged socio-economic group, while relative index of inequality measures the ratio of health outcome between the bottom and top of the socio-economic levels^(12,13)^. It should be noted that these group-based indices describe inequality across the whole population while also accounting for the size of each group and can be compared across groups if their composition remains the same. However, two important drawbacks need to be considered. (1) Researchers warn against using these indices for policy inferences as they are not appropriate for comparison if socio-economic composition of population changes across or within these groups^(13)^. For example, when education is used as a solution to improve inequality, the increase in education levels leads to people moving from the uneducated group to the educated groups, thereby changing the composition of the groups. (2) The aforementioned studies have used linear and logistic regression models to estimate the indices. As Moreno-Betancur *et al.* ^(14)^ have successfully pointed out the error in doing so, the estimation method changes with the type of parameter studied and the study design; and methodological issues such as model fit to the data become important. According to them, ideally time-to-event data should be used for such estimations and in case of cross-sectional data, negative binomial regression should be used. So far, none of the studies using slope index of inequality and relative index of inequality have explained the limitations in using them and so the practice continues.

Rank-dependent indices such as Erreygers index (EI) and Concentration index (CI) do not have these limitations. They capture all the information by ranking individuals (rather than groups) across the whole spectrum of household wealth (or mother’s education) in the study population from cross-sectional data and correlate it to levels of health^(15)^. This allows analysing even nuances due to minor differences in wealth between households (or mother’s education). As with slope index of inequality and relative index of inequality derived by regression techniques, the calculation of EI and CI allows consideration of ties in ranking between households (or mothers) in cross-sectional data. A point worth noting is that the indices are calculated differently for binary indicators (yes/no: EI) and continuous indicators (those having a range: CI). Some studies have used CI to analyse inequality for binary indicators^(8,9)^, but CI is not the right methodological choice for such bounded variables^(15)^. To our knowledge, empirical analysis of inequality in nutrition programmes using EI for binary variables and CI for continuous variables has not been done so far.

The aim of the paper is to assess whether disparities in coverage existed after intervention exposure and if the disparities were different among three countries – Bangladesh, Burkina Faso and Ethiopia. We examine the findings of large-scale maternal nutrition programmes from an equality lens using superior econometric techniques that have never been used before to our knowledge, to explore maternal nutrition inequality. The intervention package included iron and folic acid (IFA) supplementation and counselling to improve maternal IFA intake and dietary diversity. These were delivered through existing antenatal care (ANC) programmes and involved systems strengthening for improving IFA supply chains, facility, and outreach service delivery, building community demand, improving the performance of ANC providers and community health workers through training and supervision, and monitoring. No food or cash subsidies were provided. Training involved counselling skills, adherence, engaging family members and advocating for re-allocation of household budgets, promoting locally available affordable foods, and community mobilisation. Messages for PW and their husbands differed; baby and mothers’ health were the focus for PW and current and future economic costs and benefits, and feasible strategies were the focus for their husbands. Further details of the programmes and interventions can be obtained elsewhere^(1,16–18)^.

## Methods

We conducted a secondary analysis of data from cross-sectional surveys conducted at baseline and endline by the International Food Policy Research Institute (IFPRI) in Bangladesh, Burkina Faso and Ethiopia. A three-stage sampling was used in the study regions, and districts and clusters were randomly allocated to intervention and non-intervention areas. The same clusters were sampled at endline. The sample size estimation for the original analyses was done separately in each country as per baseline prevalence of IFA consumption among recently delivered women (RDW) and of dietary diversity among PW, at a power of 0·80 and a significance level of 0·05 and thus, was adequately powered to perform separate analysis in the PW and RDW populations^(16–18)^. The final sample sizes of RDW and PW are shown (Table 1).

**Table 1:** Sample sizes and survey timing by country

	Bangladesh			Burkina Faso			Ethiopia		
	Survey year	RDW	PW	Survey year	RDW	PW	Survey year	RDW	PW
Baseline	2015	2000	600	2019	1920	960	2019	344	175
Endline	2016	2000	600	2021	1920	960	2021	1889	540

RDW, recently delivered women; PW, pregnant women.

### Indicators

We calculated changes in inequality in the four indicators as described below:

- Adequate IFA consumption indicates whether RDW had consumed IFA for at least 90 days during their most recent pregnancy.
- The IFA counselling indicator measures the total number of messages received on IFA – this number differed across study countries^1^.
- Dietary diversity indicates whether a woman had consumed at least five out of ten defined food groups on the previous day.
- The binary indicator for counselling on dietary diversity indicates whether the PW received information on consuming five varieties of food from a health worker during ANC and/or home visits (also during community meetings in Burkina Faso).

### Statistical analysis

We used two equality indices after ranking the population by household wealth index score^2^ and level of mother’s education. The wealth score, a country’s study-area-specific composite index, was obtained using principal component analysis of a household’s assets, materials used in construction, and access to type of drinking water, toilet, and electricity. Since study areas were basically chosen among the disadvantaged population within each country, where rich and richest did not make much sense, the households were divided into three wealth categories (instead of using the popular categorisation) that merely demarcate the poorest from the slightly better-off.

Though wealth and education are often correlated, the logic behind analysing both wealth and education inequalities was to gain insight into the different country contexts. While study areas were basically chosen among the disadvantaged population within each country, education levels across the study countries were different. For example, the prevalence of secondary or higher education in the study population of Bangladesh was above 11 per cent while it was less than 2 per cent in Ethiopia.

The indices are EI that measures inequality of a binary indicator (such as adequate IFA consumption, maternal dietary diversity and counselling on dietary diversity) and CI that measures inequality for a continuous indicator (such as counselling on IFA consumption)^(19)^. Both indices range from –1 to +1, where the negative values indicate a pro-poor distribution, positive values indicate a pro-better-off distribution and a value of 0 indicates neutral distribution. We used ‘conindex’ in Stata 15.1 and standard errors using the cluster sandwich estimator^(20)^. The difference in indices across surveys was tested by *F*-test that assumes equal variance. An explanation of the observed difference in indices has been attempted with prevalence estimates in the supplemental figures along with the text.

## Results

Table 2 shows the population characteristics of RDW and PW at baseline for each country. In Bangladesh, three out of five RDW and PW belonged to the better-off wealth tertile, while in Ethiopia more than half the RDW belonged to the better-off wealth tertile. The proportion of PW who were adolescents was 27 per cent in Bangladesh and 13 per cent in Burkina Faso. About 12 per cent of PW had not attended school in Bangladesh, whereas this estimate was about 57 per cent in Burkina Faso. The parity distribution was balanced in Bangladesh, but the majority of RDW in Burkina Faso and Ethiopia had three or more children. Most women were housewives in Bangladesh and farmers in Burkina Faso and belonged to other occupations in Ethiopia. Bangladesh had the lowest utilisation levels of formal maternal health services at the time of the surveys. Four or more ANC visits were almost universal in Burkina Faso and Ethiopia, but the prevalence in Bangladesh was 66 per cent. The prevalence of institutional deliveries was 38 per cent in Bangladesh, 92 per cent in Burkina Faso and 77 per cent in Ethiopia. Different baseline levels of nutrition outcome indicators can be seen in adequate IFA consumption that was highest in Burkina Faso at 72 per cent and lowest in Bangladesh at 56 per cent. Similarly, mean messages on IFA counselling were also higher in Burkina Faso and Ethiopia as compared to Bangladesh. On the other hand, the prevalence of maternal dietary diversity and counselling on dietary diversity was much higher in Bangladesh as compared to the other study countries. Counselling on dietary diversity was very low in Burkina Faso at 5·63 per cent. Higher baseline levels indicate less room for improvement.

**Table 2:** Population characteristics at baseline, by country, RDW and PW

	Bangladesh				Burkina Faso				Ethiopia			
	RDW		PW		RDW		PW		RDW		PW	
	No.	Col %	No.	Col %	No.	Col %	No.	Col %	No.	Col %	No.	Col %
Household wealth												
Poorest	792	39·60	251	41·83	722	37·60	369	38·44	34	9·88	60	34·29
Less poor	620	31·00	190	31·67	631	32·86	317	33·02	114	33·14	61	34·86
Better-off	588	29·40	159	26·50	567	29·53	274	28·54	196	56·98	54	30·86
Mother’s age												
13/19	404	20·20	162	27·00	274	14·27	123	12·81	na	na	na	na
20/24	677	33·85	175	29·17	525	27·34	223	23·23	na	na	na	na
25/29 (25–50 in BF)	530	26·50	155	25·83	1121	58·39	614	63·96	na	na	na	na
30+	389	19·45	108	18·00								
Mother’s education												
Never attended school/Incomplete Grade 1	235	11·76	73	12·17	1277	66·51	643	66·98	na	na	na	na
Koranic/Literacy training	–	–	–	–	114	5·94	61	6·35	na	na	na	na
Completed Grades 1–5	703	35·17	189	31·50	307	15·99	163	16·98	na	na	na	na
Completed Grades 6–9	758	37·92	268	44·67	195	10·16	87	9·06	na	na	na	na
Completed Grades 10–12/Higher	303	15·16	70	11·67	27	1·41	6	0·63	na	na	na	na
Parity												
0	NA	NA	2	0·33	NA	NA	17	2·09	–	–	–	–
1	772	38·60	182	30·33	388	20·21	175	21·53	58	16·86	23	14·94
2	681	34·05	189	31·50	365	19·01	181	22·26	45	13·08	34	22·08
>=3	547	27·35	227	37·83	1167	60·78	440	54·12	241	70·06	97	62·99
Mother’s occupation												
Farmer	na	na	na	na	1092	56·88	539	56·15	na	na	23	14·94
Housewife	1797	89·85	530	88·33	606	31·56	317	33·02	na	na	34	22·08
Other	203	10·15	70	11·67	222	11·56	104	10·83	na	na	97	62·99
>=4 ANC visits												
No	668	33·40	NA	NA	7	0·54	NA	NA	0	0	NA	NA
Yes	1332	66·60	NA	NA	1282	99·46	NA	NA	202	100	NA	NA
Institutional delivery												
No	1238	61·90	NA	NA	150	7·81	NA	NA	79	22·97	NA	NA
Yes	762	38·10	NA	NA	1770	92·19	NA	NA	265	77·03	NA	NA

RDW, recently delivered women; PW, pregnant women; No., unweighted numbers; Col%, Column %; NA, not applicable; na, information not available.

Table 3 presents the indices on wealth inequality by nutrition indicator in intervention and non-intervention areas at baseline and endline. In intervention areas of Bangladesh, absolute wealth inequality in adequate IFA consumption became less pro-rich at endline as compared to baseline. This can be seen in the reduction in EI to almost one-third (from 0·149 to 0·052), and the change was statistically significant (for further details, see online supplementary material, Supplemental Figure 1). In Ethiopia, the CI for counselling on IFA increased from baseline to endline in non-intervention areas, and the change was significant. In intervention areas of Ethiopia, counselling on IFA became less pro-rich (CI dropped from 0·166 to 0·090), but the change was not significant (for further details, see online supplementary material, Supplemental Figure 2).

**Table 3:** Erreygers index and concentration index (ranked by household wealth), by country and indicator

	Bangladesh		Burkina Faso		Ethiopia	
	Baseline	Endline	Baseline	Endline	Baseline	Endline
Adequate IFA consumption						
Non-intervention	0·164	0·226	0·056	0·046	0·208	0·122
Intervention	0·149*	0·052*	−0·054	−0·025	0·314	0·214
Counselling on IFA^#^						
Non-intervention	0·016	0·031	0·027	0·015	0·039*	0·117*
Intervention	0·014	0·034	0·007	−0·010	0·166	0·090
Adequate dietary diversity						
Non-intervention	0·172	0·217	0·021*	0·247*	0·044	0·067
Intervention	0·283	0·136	0·144	0·166	0·040	0·162
Counselling on dietary diversity						
Non-intervention	0·065	0·028	−0·002	0·045	0·026	0·142
Intervention	−0·101	−0·019	0·004	−0·057	0·144	0·068

IFA, iron and folic acid.

^#^Concentration index used.

*Indicates statistically significant difference.

Wealth inequality in maternal dietary diversity declined in Bangladesh in intervention areas from 0·283 at baseline to 0·136 at endline, remained almost unchanged in Burkina Faso, and became more pro-better-off at endline in Ethiopia (0·040–0·162). The changes were not statistically significant except in non-intervention areas of Burkina Faso, where inequality increased significantly (for further details, see online supplementary material, Supplemental Figure 3).

Wealth inequality in receiving counselling on dietary diversity declined to one-fifth in intervention areas of Bangladesh, and it remained pro-poor (–0·101 to –0·019). In intervention areas of Ethiopia, inequality reduced to half, though it remained pro-better-off (0·144–0·068). Wealth inequality declined in intervention areas of Burkina Faso, from almost no difference across wealth categories at baseline (0·004) to favouring the poorest at endline (–0·057). However, none of the changes were statistically significant (for further details, see online supplementary material, Supplemental Figure 4).

Table 4 presents the indices on education inequality by nutrition indicator in intervention and non-intervention areas of study countries at baseline and endline. For adequate IFA consumption, EI when ranked by mother’s education was not significantly different between baseline and endline in non-intervention areas across study countries (for further details, see online supplementary material, Supplemental Figure 5). The change in education inequality in the intervention areas of Bangladesh became less pro-educated (from 0·178 to 0·070) and was significantly different. In intervention areas of Burkina Faso, education inequality for adequate IFA consumption changed from pro-low educated (–0·050) to pro-educated (0·024), and the change was significant. The CI for counselling on IFA declined significantly in the intervention areas of Bangladesh from 0·041 to 0·020 and became less pro-educated (for further details, see online supplementary material, Supplemental Figure 6).

**Table 4:** Erreygers index and concentration index (ranked by mother’s education), by country and indicator

	Bangladesh		Burkina Faso		Ethiopia	
	Baseline	Endline	Baseline	Endline	Baseline	Endline
Adequate IFA consumption						
Non-intervention	0·209	0·187	0·017	0·057	na	0·044
Intervention	0·178*	0·070*	−0·050*	0·024*	na	0·162
Counselling on IFA^#^						
Non-intervention	0·039	0·032	0·018	−0·012	na	0·107
Intervention	0·041*	0·020*	0·005	−0·005	na	0·063
Adequate dietary diversity						
Non-intervention	0·223	0·179	0·018	0·025	na	0·126
Intervention	0·258*	0·052*	−0·007	−0·019	na	0·076
Counselling on dietary diversity						
Non-intervention	0·092	−0·051	0·003	0·054	na	0·096
Intervention	0·022	0·003	0·026	0·064	na	0·117

IFA, iron and folic acid.

^#^Concentration index used.

*Indicates statistically significant difference.

A significant drop in education inequality for dietary diversity was observed from baseline (0·258) to endline (0·052) in the intervention areas of Bangladesh where it became less pro-educated (for further details, see online supplementary material, Supplemental Figure 7). For counselling on dietary diversity, there was no significant change in education inequality between baseline and endline in non-intervention and intervention areas in any of the study countries (for further details, see online supplementary material, Supplemental Figure 8).

## Discussion

Overall, the study shows that there was no generalised shift towards greater equality in intervention areas, even though the overall prevalence of indicators was higher in intervention areas. Yet, although improving equality was not an explicit objective of the programmes, some maternal nutrition indicators were trending towards lower inequality after exposure to nutrition interventions. To our knowledge, this is the first study to explore changes in inequality in maternal nutrition service delivery and uptake of practices by PW following intervention programmes. The study is unique in assessing inequality using pre-test–post-test randomised intervention and control group evaluation data from low- and middle-income countries. Though no attempt has been made to imply causality, the strong study design allows our conclusion regarding the possible inequality lowering effect of nutrition interventions to be potentially valid and worth pursuing. We examined inequality by PW’s education in addition to wealth; the study therefore also adds to the literature by providing empirical evidence on education inequality.

As summarised in Table 5, statistically significant reductions in education inequality were observed for adequate IFA consumption, counselling on IFA and dietary diversity in intervention areas of Bangladesh, and for adequate IFA consumption in intervention areas of Burkina Faso. While wealth inequality in adequate IFA consumption decreased over time in the intervention areas in all three countries, it increased for counselling on IFA in Bangladesh and Burkina Faso. This indicates the need for more targeted studies on barriers to counselling services among the less advantaged PW. The lack of alignment in results for IFA consumption and IFA counselling in Bangladesh and Burkina Faso suggests that IFA consumption cannot be attributed only to IFA counselling. This is consistent with studies on factors influencing IFA intake^(21)^.

**Table 5:** Summary table showing change in inequality (absolute) in maternal nutrition indicators after programme exposure in non-intervention and intervention areas

		Bangladesh		Burkina Faso		Ethiopia	
Indicators	SE variable	Non-Int.	Int.	Non-Int.	Int.	Non-Int.	Int.
Recently delivered women							
Adequate IFA consumption	Wealth	Increased	Reduced*	Reduced	Reduced	Reduced	Reduced
	Education	Reduced	Reduced*	Increased	Reduced*		
Counselling on IFA^#^	Wealth	Increased	Increased	Reduced	Increased	Increased*	Reduced
	Education	Reduced	Reduced*	Reduced	No change		
Pregnant Women							
Dietary diversity	Wealth	Increased	Reduced	Increased*	Increased	Increased	Increased
	Education	Reduced	Reduced*	Increased	Increased		
Counselling on dietary diversity	Wealth	Reduced	Reduced	Increased	Increased	Increased	Increased
	Education	Reduced	Reduced	Increased	Increased		

SE variable, socio-economic variable; Non-Int., non-intervention; Int., intervention.

# Concentration index used.

* indicates statistically significant difference.

Wealth equality declined significantly at endline in the non-intervention group for counselling on IFA in Ethiopia and dietary diversity in Burkina Faso, but not in the intervention group. This may be considered a protective effect of the interventions as the period between baseline and endline was marked by COVID-19 disruptions in Burkina Faso and Ethiopia. For dietary diversity and counselling on dietary diversity, variable results were observed in inequality by wealth and education. For example, during the programme implementation period, inequality declined in Bangladesh while it increased in Burkina Faso and Ethiopia. The timing of COVID-19 coincided with the intervention period in Burkina Faso and Ethiopia but not in Bangladesh^(16,17,22)^. In addition, the much poorer maternal education in Burkina Faso as compared to Bangladesh might have played a role.

Though the same package of globally recommended interventions was implemented in all three countries, adapting it to existing health systems and local contexts resulted in variations. This and external factors, such as disruptions due to COVID-19 and content of some indicators, were likely explanatory factors for the differing results across countries. Baseline variations among the countries could have limited the potential to improve the prevalence of nutrition indicators where prevalence levels were already high. Since the nutrition interventions were integrated into ANC services, issues with inequality in ANC at baseline may reflect the underlying ANC service delivery platforms as noted below.

First, the structures of ANC platforms varied from a community-based NGO programme in Bangladesh to a primary health centre-based government programme in Burkina Faso, and a primary health centre plus health post-based government programme in Ethiopia. With regard to functioning, the Bangladesh model used a ‘performance improvement cycle’ to build nutrition service provision more comprehensively, while the Burkina Faso model focused primarily on health systems strengthening to improve ANC services with linkages to community health agents. The Ethiopia model focused on capacity building of selected health systems components, including supplies, monitoring, and ANC providers at primary health centre hubs and in health posts along with imparting key messages through group sessions and home visits by health extension workers and volunteers^(1)^.

Second, economic constraints and geographic barriers were addressed differently. In Bangladesh, IFA tablets were made free in intervention areas and female community health volunteers delivered IFA and counselling to the PWs’ doorsteps^(1)^. On the other hand, though IFA tablets and facility visits were made free in both Burkina Faso and Ethiopia and services were brought nearer to the community in Ethiopia by permitting health extension workers to deliver ANC services at the health post level, PW were still required to visit health facilities to collect them and receive counselling, thereby benefitting only those women who had time, resources, and family support to make facility visits.

Third, community agents in Burkina Faso and Ethiopia were remunerated through fixed monthly salaries and were not linked to coverage or quality criteria. In contrast, community volunteers in Bangladesh were provided performance-based cash incentives, with clearly structured criteria including reaching as many PW as possible. This may have motivated community volunteers to make extra efforts in reaching distant and less receptive households.

Fourth, in Bangladesh, additional special forums were organised for husbands of PW through outreach sessions at convenient sites and at times convenient for them. Husbands played a pivotal role in each country in facilitating PW attendance at ANC sessions and in the uptake of PW’s IFA consumption and dietary diversity. Similar forums were not successful in Burkina Faso because the staff found it difficult to identify the right venues to reach husbands of PW consistently with high coverage. In Ethiopia, no special gatherings for husbands were used; instead, home visits were made and other family members present in the household were engaged.

As noted above, despite a common package of interventions, the strategies used for service delivery differed and may account for more reductions in inequality in Bangladesh as compared to Burkina Faso and Ethiopia^(7,23)^. While Tables 3–5 show that inequality in Bangladesh reduced in intervention areas at endline as compared to baseline, Figures 1–8 in the supplemental material show how this came about. The prevalence among the poorest increased to almost the same extent as the better-off. While the first three bars representing prevalence at baseline and non-intervention areas at endline remained almost the same, the increase in prevalence (fourth bar) in intervention areas at endline is drastic. This change is higher in the poorest section as compared to the less poor and better-off. Our results align with an analysis of eighteen RMNCH indicators in thirty-six low-income and middle-income countries that found community-based interventions had a more equitable distribution and those primarily delivered in health facilities did not^(7)^. Similarly, past research suggests that ANC attendance in facilities and high-quality ANC services tend to favour the better-off^(24)^. A review of counselling services in South Asian countries found that poor households and those utilising lower level or public health facilities were less likely to receive counselling than wealthier households or those utilising higher levels of government facilities or private facilities^(25)^.

COVID-19 disruptions also had a role in the Burkina Faso and Ethiopia results. The epidemic took a heavy toll on the delivery and utilisation of ANC due to interrupted services in Ethiopia and on household food security due to high food prices in Burkina Faso and Ethiopia during programme implementation^(26)^. In contrast, maternal nutrition interventions in Bangladesh were implemented and evaluated during 2015–2016, preceding COVID-19 restrictions, and were conducted following a period of economic growth and overall food availability^(27)^. The incorporation of multiple components in the community-based intervention was a successful strategy deployed by Bangladesh, as well^(28)^.

The type of PW behaviour also had a bearing on the study results, with IFA consumption responding to interventions better than dietary diversity. Relatively greater effort and costs were likely to have been incurred in improving dietary diversity at the household and individual levels, for example, for shifting embedded gender norms and perceptions of family members to increase specified foods and meals for PW, while IFA was available free of cost and could be obtained through public health services. For service delivery costs, ensuring IFA supplies and improving health providers’ IFA distribution and counselling performance are likely to require less effort as compared to efforts to alter household budgets and food allocation for PW.

### Limitations

The study results are subject to the following limitations. (1) Absence of information on survey weights prevented significance testing in changes (difference-in-difference) in inequality indices. Instead, a cluster sandwich estimator was used. (2) Though the simplistic conceptualisation of counselling on dietary diversity did not do justice to the full contents of counselling that also covered food quantity and meal frequency and tailoring of messages to individual client needs, it was an indication of efforts made to modify PW diets. In addition, the conceptualisation disregards other factors that influence the quality of counselling including (but not limited) to training, mentoring and counselling materials. In future, better measures of counselling quality need to be developed. (3) In Bangladesh, education inequality used education categories for ranking because of a lack of yearly information on mother’s education. It has been suggested that multiple ties in the fractional ranking of the education variable may be problematic^(29)^, but the Stata command ‘conindex’ takes care of these ties in computing the point estimate while the ‘cluster’ option takes care of ties between non-independent observations^(30)^. (4) In Ethiopia, there was the absence of consolidated information on the total number of IFA consumed in baseline datasets of RDW and on education in baseline datasets of both RDW and PW datasets, so only endline was used. (5) Note that some estimates are based on sample sizes as low as 86 (Ethiopia PW baseline data). Though there are no guidelines on sample sizes and index calculations are based on cumulative means and do not require large sample sizes, estimate precision increases with larger samples. (6) It should be noted that dividing household wealth into three equal-sized groups or wealth tertiles incorporates only the concept of relative poverty because most of the respondents belonged to deprived sections of society in rural areas where the programmes were implemented.

### Conclusions

The results from three different country and programme contexts provide useful insights into programmatic strategies needed to improve inequality in maternal nutrition interventions which can be helpful in other programmes. Although the programmes were not explicitly designed to reduce inequality, it appears that certain elements of policies and interventions helped in improving inequality^(23,31,32)^. Pro-poor characteristics of the long-standing community-based MNCH programme in Bangladesh transferred easily to the newly integrated nutrition components; the type of remuneration, competence, experience and commitment of community workers enabled them to more effectively reach the poorest and motivate behaviour change among PW in Bangladesh. This aligns with past research^(33)^ and reinforces the WHO guidance on maintaining community health worker programmes to maximise coverage^(34)^ and foster equity^(35)^. However, this analysis indicates that more needs to be done to explicitly identify and reduce inequality.

Applying inequality analysis across three countries with different contexts provides further insight into the role of health systems’ infrastructure and use of rigorously designed programme interventions as compared to routine intervention packages. The study interventions tailored global recommendations^(36)^ to each context and aimed at improving overall coverage and adoption of nutrition practices, but they were not aimed specifically at reducing inequalities. Large-scale programmes usually focus on improving coverage or usage but do not usually have the objective of reducing inequality. Through this study, we examined whether this approach needs to be enhanced through the explicit introduction of equality strategies. Since inequality is a major hurdle in achieving SDGs, the programme insight gained from this article will be helpful to programmers in future.

According to the 2030 collaboration, reducing inequalities within countries at a faster pace could be one of the ways to achieve SDGs by 2030^(37)^. The main takeaways from this study for nutrition programmes to help countries achieve these goals are as follows: (a) assessing inequality issues early while designing interventions through formative studies, (b) monitoring inequality indicators throughout implementation, (c) diligently addressing inequality through targeted interventions, setting aside resources to address inequality and motivating frontline workers to reach left-out communities and areas, (d) motivating managers and providers to build community linkages and engage family members to drive services, coverage and quality closer to where the disadvantaged reside and (e) making inequality analysis a routine part of impact evaluations.

## Supporting information

Godha et al. supplementary material Godha et al. supplementary material

## Data Availability

De-identified participant data used for generating the results are accessible through https://www.aliveandthrive.org/en/our-data, linked to https://dataverse.harvard.edu/dataverse/harvard.

## Footnotes

1 This is because messages and contact points were tailored to each country context. Messages were received during both ANC and counselling sessions at home visits in Bangladesh; at three different contact points in Burkina Faso: during ANC visits in facilities, home visits and community meetings/GASPA (local term for women’s support groups); and at two different contact points in Ethiopia: during ANC visits in health facilities and home visits. The country specific message list is as follows:

- *Bangladesh*: Take 1 tablet daily; IFA prevents anemia; IFA reduces the risk of LBW; IFA reduces the risk of maternal death; do not drink tea/coffee with IFA; and others.
- *Burkina Faso*: Take 1 tablet daily; take 180 IFA tablets from the fourth month of pregnancy until delivery; IFA prevents anemia; IFA reduces the risk of LBW; IFA reduces the risk of maternal death; do not to take tea/coffee with IFA; take IFA tablets with water before sleeping at night; eat more fruits and vegetables daily when taking IFA tablets; take two IFA tablets per d/take IFA with or after meals; prevent or manage side effects (only endline) and others.
- *Ethiopia* : Take 180 IFA tablets daily during pregnancy; take one IFA tablet per d from the fourth month of pregnancy; IFA prevents anemia; IFA reduces the risk of LBW; IFA reduces the risk of maternal death; not to take tea/coffee with IFA; take IFA tablets with water or lime juice at night; potential side effects; and how to manage those side effects.

2 The wealth index was created using principal component analysis. Variable selection and creation of wealth index has been conducted separately for each country, but it remains the same within a country across non-intervention and intervention areas as well as baseline and endline surveys. Further details are available from the authors.

## Supplementary material

For supplementary material accompanying this paper visit https://doi.org/10.1017/S1368980024001150
