# Supplementary material for: Inequality in iron and folic acid consumption and dietary diversity in pregnant women following exposure to maternal nutrition interventions in three low- and middle-income countries
Source: Public Health Nutr. 2024 May 24;27(1):e149. doi: 10.1017/S1368980024001150 (PMC11617414; doi:10.1017/S1368980024001150)
Supplement: Godha et al. supplementary material [file S1368980024001150sup001.pdf]

Supplemental Material

Table 3 can be further explained by the prevalence of adequate IFA consumption across the wealth categories in each country as shown in bar-graphs (Figure 1). The first and third columns show the prevalence of adequate IFA consumption in the non-intervention areas at baseline and endline for each country respectively. The asterisks indicate a significant difference across the four groups for wealth categories.

In Bangladesh, the prevalence at endline (as compared to baseline) was lower among the poorest and less poor but higher among the better-off PWs. This resulted in an increase in inequality in the non-intervention areas from .164 to .226. In intervention areas of Bangladesh, the prevalence of adequate IFA consumption at baseline and endline (represented by the second and fourth columns) increased across all three wealth categories. This occurred in a declining manner with a change of 33, 30, and 23 percentage points among the poorest, less poor, and better-off wealth categories. Even though the prevalence of adequate IFA consumption was higher in the better-off groups, the greater change among the poorest as compared to their wealthier counterparts led to a huge and significant reduction in inequality.

In Burkina Faso, the baseline prevalence of adequate IFA consumption in non-intervention and intervention areas remained between 69-74 percent across wealth categories. It was the highest among the poorest in non-intervention areas leading to a negative EI and was the highest among the better-off in intervention areas leading to a positive EI. The change over time was between 4-5 percentage points across the wealth tertile in non-intervention areas and show almost no change in the EI. In intervention areas at endline, the change was between 9 to 17 percentage points and the prevalence among the poorest was higher than among the better-off. This explains the improvement in EI which still remained pro-poor. (Table 3)

In case of Ethiopia, baseline data on IFA consumption was not available. The asterisks in the graph indicate significant difference for a category between non-intervention and intervention areas at endline. The prevalence of adequate IFA consumption in non-intervention areas at endline was the lowest among the poorest and highest among the better-off leading to an EI of .122. The prevalence of adequate IFA consumption in intervention areas at endline was higher than in non-intervention groups across all three wealth tertile. However, while the

difference between the poorest and better-off in non-intervention areas was about 13 percentage points, in intervention areas it was about 20 percentage points. This explains the higher EI of .214 in intervention areas at endline, indicating greater inequality; the differences were not statistically different. (Table 3)

Supplemental Figure 1. Prevalence of adequate IFA consumption, by household wealth and country

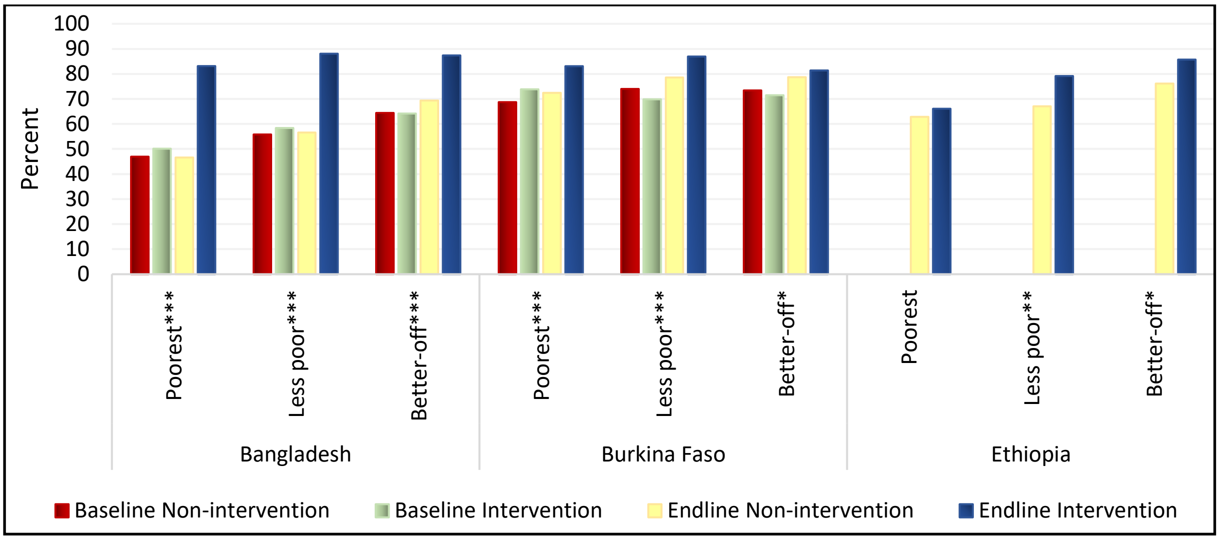

** p-value<=0.05; ** p-value<=0.001; *** p-value<=0.000*

Figure 2 shows that the mean number of messages did not increase much in the intervention areas of Bangladesh from baseline to endline and it was a graded increase from poorest to better-off whereas in non-intervention areas it decreased across wealth categories except the Better-off. The difference between the poorest and better-off increased in both non-intervention and intervention areas from baseline to endline and was more than double. Hence the increase in inequality though it was not a significant change. The low number of messages reflects the counseling approach used in Bangladesh that focused on a few key messages to avoid overwhelming health workers and recipients of messages. (Table 3)

In Burkina Faso, there was a large increase in intervention areas in the mean number of messages received on IFA from baseline to endline, but the change was not statistically significant. In Ethiopia, the change in non- intervention areas was large but resulted in a higher increase in the better-offer sub-populations leading to an increased CI that was significantly different from baseline. (Table 3)

Supplemental Figure 2. Counselling on IFA among RDW, by household wealth and country

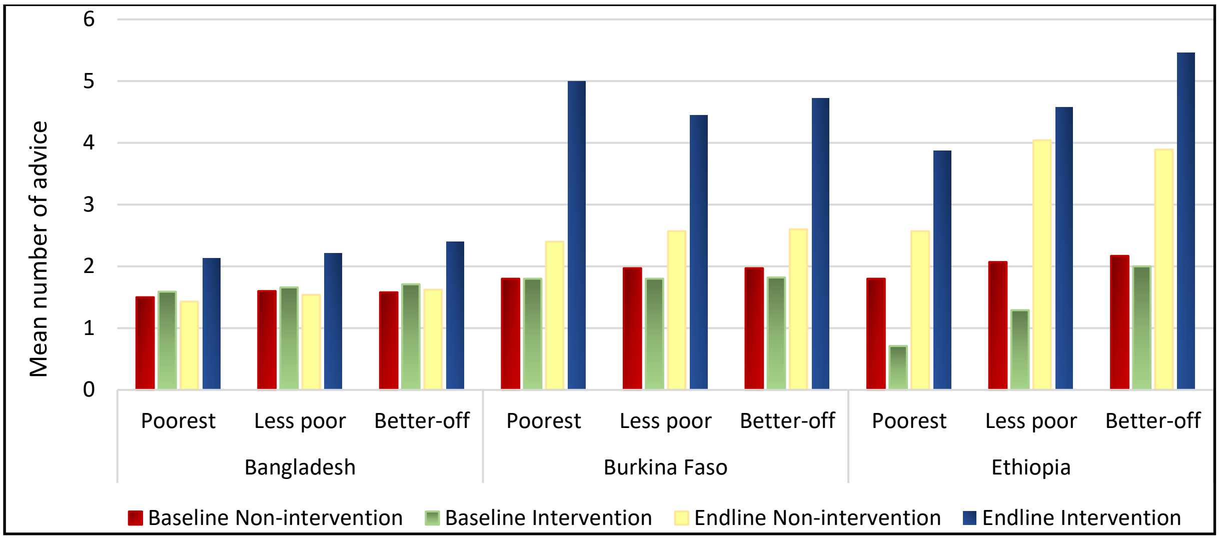

As shown in Figure 3, in the non-intervention areas of Burkina Faso, the prevalence of dietary diversity dropped by 7-9 percentage points among the poorest and less poor while it increased significantly by 15 percentage points among the better-off. While the dietary diversity prevalence was higher among the poorest as compared to the better-off at baseline, the opposite was true by endline with a difference of 24 percentage points. This explains the significant rise in inequality in the non-intervention areas of Burkina Faso. (Table 3)

Supplemental Figure 3. Prevalence of dietary diversity among PW, by household wealth and country

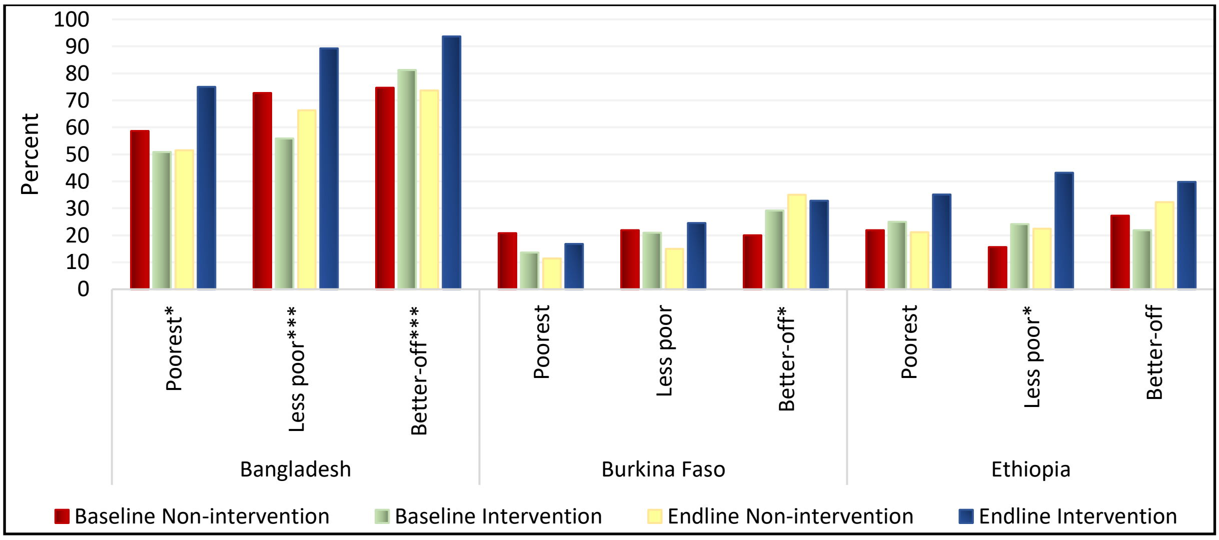

** p-value<=0.05; ** p-value<=0.001; *** p-value<=0.000*

The findings from Table 3 are explained by the fact that even though the prevalence of counselling on dietary diversity rose from baseline to endline, it resulted in evening out the distribution across wealth categories. Note the universal coverage across wealth sub-groups in intervention areas of Bangladesh at endline (Figure 4).

Supplemental Figure 4. Prevalence of counselling on dietary diversity among PW by household wealth and country

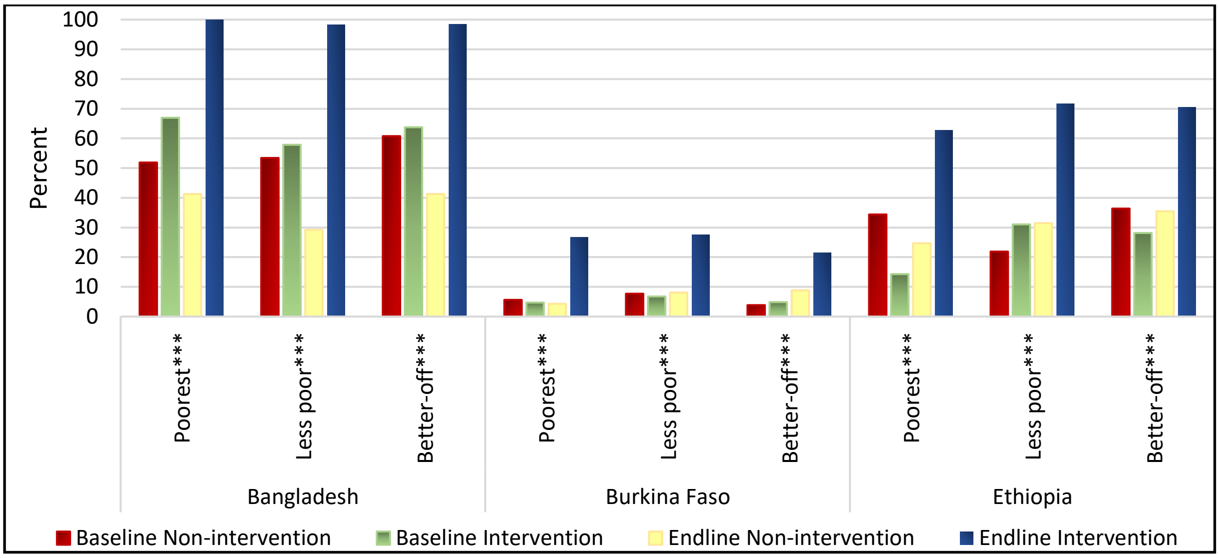

** p-value<=0.05; ** p-value<=0.001; *** p-value<=0.000*

The prevalence of adequate IFA consumption by education categories in Figure 5 explains the inequality results in Table 4. In Bangladesh, the prevalence was almost the same in baseline non-intervention and intervention areas and endline non- intervention areas. However, a graded increase in prevalence is observed with increasing education. The prevalence of adequate IFA consumption in intervention areas at endline is much higher than the other three areas but a graded decline in increment is observed by increasing education. The difference in prevalence across the least and most educated women in non-intervention areas was around 37 percentage points at baseline and remained the same at endline which explains an EI of.21 and .19 respectively. In intervention areas, this difference decreased from 29 percentage points at baseline to around 9 percentage points at endline. This explains the statistically significant drop in EI in intervention areas from .18 at baseline to .07 at endline.

In Burkina Faso, the prevalence of adequate IFA consumption in non-intervention areas across education categories was almost similar at baseline lying between 69-74 percent and at

endline between 75-80 percent, except among the most educated where it remained the highest. The change was lowest among the least educated sub-population while it was higher among the more educated thereby explaining the increase in EI from .017 at baseline to .057 at endline. In intervention areas, the prevalence of adequate IFA consumption at baseline was between 63-74 percent across education categories with the maximum difference between categories being 9 percentage points. By endline, the prevalence of adequate IFA consumption had increased and was between 83-88 percent across education categories with the maximum difference between categories being 5 percentage points. This explains the significant drop in EI in intervention areas.

Supplemental Figure 5. Prevalence of adequate IFA consumption, by mother's education and country

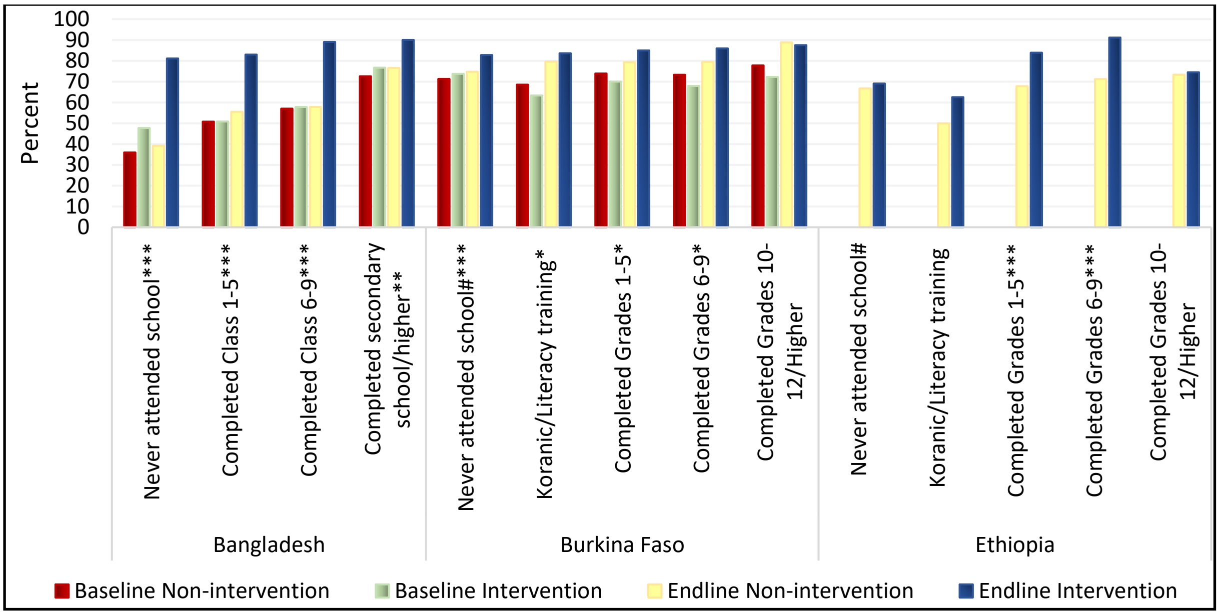

** p-value<=0.05; ** p-value<=0.001; *** p-value<=0.000*

In the intervention areas of Bangladesh (Table 4), the mean number of messages on IFA at baseline was slightly higher among the more educated (Figure 6). By endline, the mean number of messages had risen across all education sub-groups. The biggest difference between two categories was 0.47 at baseline which had dropped to 0.42 at endline leading to a drop in CI.

Supplemental Figure 6. Counselling on IFA among RDW, by mother's education and country

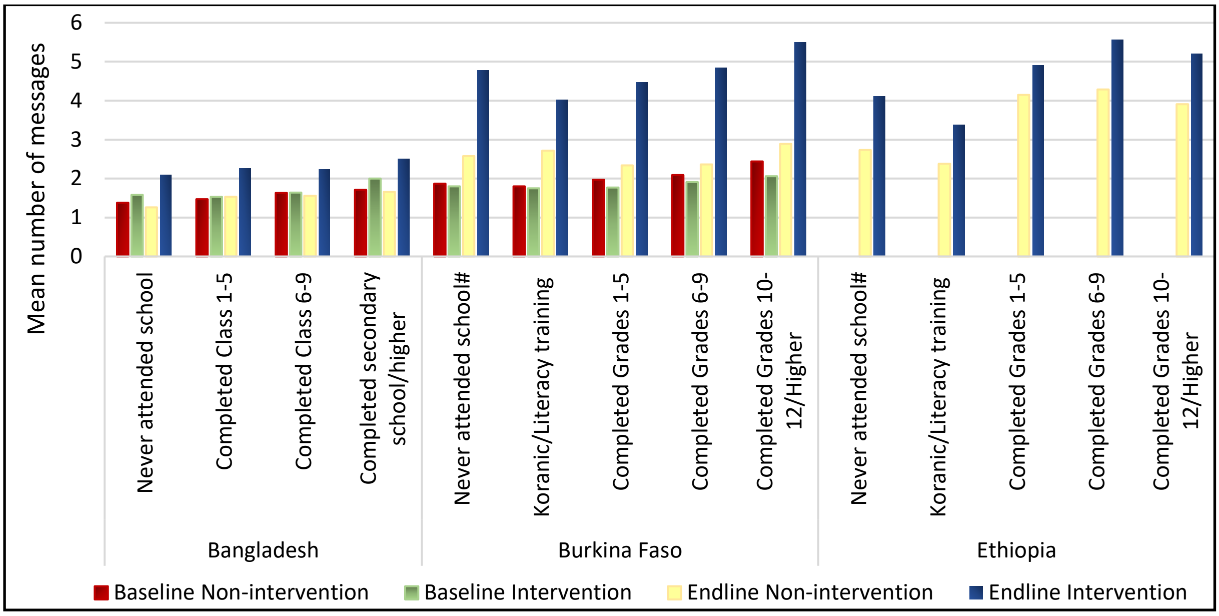

Figure 7 shows that the prevalence of dietary diversity rose in the intervention areas of Bangladesh across all education categories. But the rise was disproportionately higher among the lesser educated with a gradient decline with increasing education. This resulted in the difference in prevalence between the two extreme education categories from 46 percentage points at baseline to 12 percentage points at endline. Such a huge change brought about a more balanced distribution of dietary diversity across the education categories resulting in reduced inequality.

Supplemental Figure 7. Prevalence of dietary diversity among PW by mother's education and country

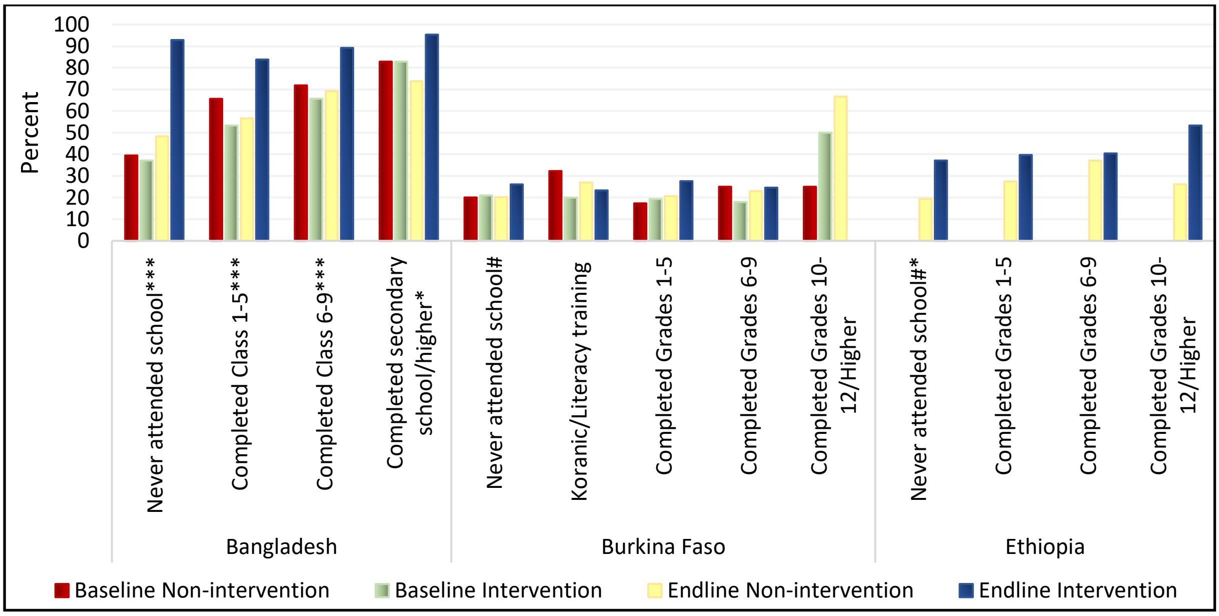

** p-value<=0.05; ** p-value<=0.001; *** p-value<=0.000*

In Ethiopia, though the prevalence of counselling on dietary diversity was higher by 30-47 percentage points in intervention areas as compared to non-intervention areas at endline, the prevalence was higher among the higher educated (Figure 8). This explains the EI in the two areas at endline in the country. Note the universal coverage across education sub-groups in intervention areas of Bangladesh at endline and the comparatively low prevalence in Burkina Faso.

Supplemental Figure 8. Prevalence of counselling on dietary diversity among PW, by mother's education and country

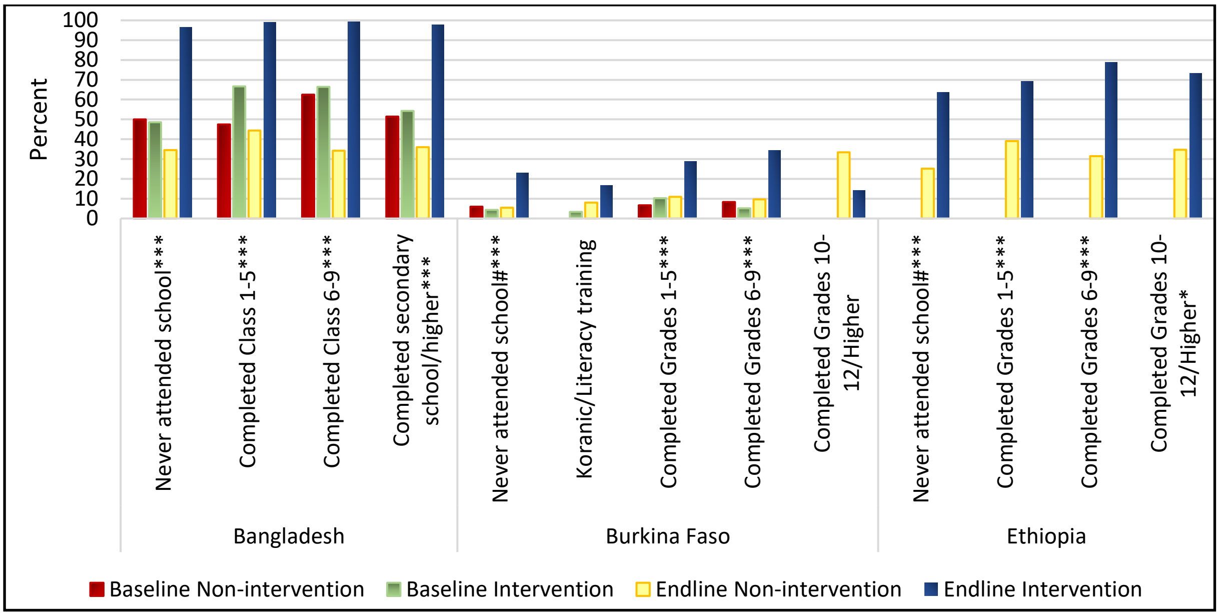

** p-value<=0.05; ** p-value<=0.001; *** p-value<=0.000*
